# Sustainable Carbon Source from Almond Shell Waste: Synthesis, Characterization, and Electrochemical Properties

**DOI:** 10.3390/ma19010008

**Published:** 2025-12-19

**Authors:** Katarina Nikolić, Milan Kragović, Marija Stojmenović, Jasmina Popović, Jugoslav Krstić, Janez Kovač, Jelena Gulicovski

**Affiliations:** 1 “VINČA” Institute of Nuclear Sciences, National Institute of the Republic of Serbia, University of Belgrade, Mike Petrovića Alasa 12-14, Vinča, 11351 Belgrade, Serbiam.kragovic@vin.bg.ac.rs (M.K.); mpusevac@vin.bg.ac.rs (M.S.); 2 Faculty of Forestry, University of Belgrade, Kneza Višeslava 1, 11000 Belgrade, Serbia; jasmina.popovic@sfb.bg.ac.rs; 3 Centre for Catalysis and Chemical Engineering, National Institute, Institute of Chemistry, Technology and Metallurgy, University of Belgrade, Studentski Trg 12-16, 11001 Belgrade, Serbia; jugoslav.krstic@ihtm.bg.ac.rs; 4 Department for Surface Engineering, Institute “Jožef Stefan”, Jamova Cesta 39, 10000 Ljubljana, Slovenia; janez.kovac@ijs.si

**Keywords:** almond shell, biowaste carbon, carbon paste electrode (CPE), electrochemical sensors

## Abstract

This study demonstrates the complete transformation of almond shell waste into a high-performance carbon material for carbon paste electrode (CPE) fabrication. The biocarbon was synthesized via carbonization at 800 °C and subsequently activated with CO_2_, resulting in a semicrystalline structure rich in carbonyl groups—consistent with its lignocellulosic origin (34.25% cellulose, 13.48% hemicellulose, 48.03% lignin). Carbonization increased the total pore volume of carbonized almond (CAR_ALD) by nearly 13-fold and the specific surface area by over two orders of magnitude compared to raw almond (RAW_ALD), while CO_2_ activation further enhanced activated almond’s (ACT_ALD) surface area (~19%) and pore volume (~35%). To improve electrochemical performance, Bi_2_O_3_ doped with Sm was applied as a surface modifier. Comprehensive characterization (N_2_ physisorption X-Ray Diffraction (XRD), Fourier Transform Infrared Spectroscopic Analysis (FTIR), X-Ray Photoelectron Spectroscopic Analysis (XPS), Thermogravimetric and Differential Thermal Analysis (TG-DTA), Cyclic voltammetry (CV), Electrochemical impedance spectroscopy (EIS)) confirmed the material’s structural integrity, graphitic features, and successful modifier incorporation. Electrochemical testing revealed the highest current response (48 µA) for the CPE fabricated from CAR_ALD/Bi_2_O_3_-Sm, indicating superior electrocatalytic activity and reduced charge transfer resistance. Notably, this is the first report of a fully functional CPE working electrode fabricated entirely from waste material.

## 1. Introduction

The increasing environmental challenges, driven by global population growth and the rapid depletion of natural resources, have intensified the search for more sustainable and circular approaches to resource management and the economy. Environmental challenges refer to issues such as climate change, pollution, deforestation, and loss of biodiversity, which threaten ecosystems and human well-being. In this context, alternative, non-wood biobased materials have emerged as a promising strategy, particularly those derived from underutilized or discarded biomass sources [1,2].

Biomass refers to organic material of biological origin, such as agricultural residues, agro-industrial by-products, forestry waste and dedicated energy crops, which can be transformed into valuable products or energy sources. One of its greatest strengths lies in its renewability and potential to replace fossil-based resources across various applications. Depending on its origin and processing, biomass can serve as a raw material for energy production (biofuels, biogas), the synthesis of chemicals and materials, or the development of sustainable packaging composites and environmental remediation tools [3,4,5,6]. By redirecting attention toward residues that would otherwise contribute to environmental burdens, biomass valorization not only reduces waste but also presents a strategic opportunity to create sustainable materials and foster regional bioeconomies [7].

Agricultural and agro-food residues are among the most promising sources of biomass, often produced in large quantities but still underused [8]. One key type of these residues is nut shells—hard outer coverings of fruits like almonds, walnuts, hazelnuts, peanuts, and pecans. Nuts are processed worldwide due to their high nutritional value, being rich in unsaturated fatty acids, proteins, fibers, and antioxidants. However, this large-scale processing produces significant amounts of shell waste, which is frequently ignored [9]. Recently, nut shells have gained recognition as a valuable biomass feedstock because of their abundance, low cost, and high lignocellulosic content [10]. Walnut shells, for example, are widely used to produce activated carbon with high surface area and microporosity, effective for removing heavy metals and organic pollutants from water [10,11,12]. Similarly, hazelnut shells show potential as precursors for porous carbon electrodes in supercapacitors and lithium-ion batteries, thanks to their excellent carbon yield and electrochemical stability [13,14]. Pistachio shells have shown promise in developing solid acid catalysts and bio-based composites [15], while pecan shells have been studied for pharmaceutical applications, such as natural excipients or carriers in drug delivery [16,17,18,19].

Among nut species, almonds (*Prunus dulcis*) are widely cultivated, with notable prevalence in Mediterranean climates and arid areas [7]. While the almond industry primarily focuses on producing edible kernels, it also generates substantial amounts of shells and husks, which are often regarded as low-value waste or used for inefficient energy applications [7]. In addition, almond shells possess exceptional physicochemical properties, including a high fixed carbon content, a dense lignocellulosic matrix, and natural porosity, which make them ideal precursors for carbon-rich materials, particularly in thermochemical conversion processes such as pyrolysis or activation [20]. Due to their structural rigidity and thermal stability, almond shells undergo controlled carbonization and functionalization, enabling the development of materials with tailored surface area, porosity, and surface chemistry [21]. Consequently, they have been effectively utilized in various applications, including electrochemical energy storage [22], catalysis, adsorption [23], and environmental remediation [24].

In recent years, electrochemical techniques using carbon-based materials have attracted considerable attention for the qualitative and quantitative analysis of various types of pollutants. While spectroscopic and chromatographic methods, such as high-performance liquid chromatography (HPLC), inductively coupled plasma optical emission spectrometry/mass spectrometry (ICP-OES/MS), and atomic absorption spectrometry (AAS), are highly reliable and accurate, they require sophisticated, costly instrumentation, lengthy analysis times, and complex sample preparation [24]. In contrast, electrochemical methods offer a faster, cost-effective alternative with minimal sample preparation. Additionally, their portability enables real-time analysis [25].

A standard electrochemical cell comprises three electrodes: the working electrode, the reference electrode, and the counter electrode. The choice of technique—voltammetric or amperometric—can markedly enhance the precision and selectivity of measurements [26]. Among these, the working electrode is pivotal to the cell’s overall performance. Historically, mercury electrodes were commonly employed; however, due to their toxicity, they have been largely supplanted by safer alternatives, with carbon-based electrodes now being the most prevalent.

Several types of carbon electrodes are now in use, including glassy carbon, carbon paste, screen-printed, and laser-induced carbon electrodes [27].

Carbon is an abundant and cost-effective material for electrode fabrication. It offers a wide potential window, is non-toxic, and is easy to handle. Moreover, its ability to form covalent bonds with various functional materials enables surface modifications that enhance the electroactive surface area and active site density, thereby improving selectivity, precision, and accuracy in electrochemical analyses. Numerous studies have explored the use of metal and non-metal oxides, polymers, and different allotropic forms of carbon as modifiers, leading to high-performance sensors with proven effectiveness in environmental monitoring [28,29]. In recent years, nanomaterial-based modifiers doped with rare-earth elements, such as samarium, have gained increasing attention. These materials have demonstrated excellent performance in the development of various electrode systems and have significantly enhanced the functional properties of the resulting structures. Consequently, they have contributed to notable improvements in applications related to batteries, fuel cells, electroanalysis, and other applications [30,31,32,33,34].

Recently, biochar and carbonized biomass materials (biocarbons) have emerged as sustainable and recyclable alternatives for electrochemical sensor development. The working electrode can be fabricated using commercial graphite and biocarbon in varying mass ratios. Researchers have successfully integrated different biocarbon sources, such as walnut shells (15%), barley (15%) [35,36], Platanus seeds (21.4%) [37], coconut shells (30%) [38], rice straw (40%) [39], and maize tassel (70%) [2]. As research in this area continues, the quest for simple, cost-effective electrode designs with high sensitivity and selectivity remains an ongoing challenge.

In that context, this research presents synthesis of carbon material derived from waste almond shells, marking the first instance of a working electrode fabricated entirely from waste-derived biocarbon. Almond shells were selected due to their wide availability and independence from climatic and geographical constraints. The biocarbon, obtained through carbonization, was characterized using various physicochemical techniques (N_2_ physisorption, X-ray diffraction analysis (XRD), Fourier transform infrared spectroscopic analysis (FTIR), X-ray photoelectron spectroscopic analysis (XPS) and Thermogravimetric and differential thermal analysis (TG-DTA)) to analyze its structural and material properties. To enhance the electrochemical performance of the biocarbon, bismuth oxide, both in its pure form and doped with 5 wt% samarium, was synthesized and used as a functional modifier. The prepared materials were then employed in the fabrication of a working electrode composed entirely of waste-derived carbon, demonstrating a novel approach in electrochemical sensor development. The electrochemical behavior of this innovative biocarbon-based electrode was evaluated using cyclic voltammetry (CV) and electrochemical impedance spectroscopy (EIS), confirming its feasibility for electrochemical applications.

## 2. Materials and Methods

### 2.1. Materials

Almond shell (RAW_ALD) was used as a raw material to obtain the carbonized material. Almond shells were collected from a household near Belgrade, Serbia, washed with distilled water, dried at 105 °C for 24 h, and then ground to a powder with a particle size of less than 50 µm. The other chemicals used in this study were Bi(NO_3_)_3_·5H_2_O (Kemika, Zagreb, Croatia), Sm(NO_3_)_3_·6H_2_O (Aldrich, Darmstadt, Germany), HNO_3_ 65% and NaOH (Lamcher, Brno, Czech Republic), ethanol 96% (Reachem, Novi Sad, Serbia) and citric acid monohydrate (Centrohem, Stara Pazova, Serbia). All chemicals were analytical grade and used without any purification treatment.

### 2.2. Synthesis of Carbon Material, Bi_2_O_3_, Bi_2_O_3_-Sm and Preparation of Working Electrode

#### 2.2.1. Synthesis of Bi_2_O_3_

The synthesis of Bi_2_O_3_ was performed following the work of Hormigos et al. [40] with slight modifications. Briefly, particles of Bi_2_O_3_ were synthesized by mixing two solutions of 20% Bi(NO_3_)_3_ in 0.05 M HNO_3_ and 13% NaOH under vigorous stirring at a magnetic stirrer. The mixture was heated at 90 °C for 2 h under continuous stirring. The particles were collected by filtration, then washed with distilled water and ethanol, and dried at 60 °C overnight. The resulting particles, Bi_2_O_3,_ were sintered for 2 h at 800 °C.

#### 2.2.2. Synthesis of Bi_2_O_3_-Sm

The next synthesis was described by Faraz et al. [41]. In 100 mL of distilled water, Bi(NO_3_)_3_·5H_2_O and citric acid were dissolved in a molar ratio of 1:1, and 5% mass of Sm from Sm(NO_3_)_3_·6H_2_O was added. The mixture was homogenized on a magnetic stirrer. The resulting solution was heated up to 150 °C on a magnetic stirrer until the solution was converted to gel form. The heat was continued until the gel ignited, forming a powdery precipitate. The resulting particles, Bi_2_O_3_-Sm, were transferred to a furnace and annealed for 2 h at 600 °C.

#### 2.2.3. Synthesis of Carbon Materials

RAW_ALD powder (42 g) was weighed and loaded into a steel vessel for carbonization. The procedure was performed in a horizontal stainless-steel tube furnace equipped with an automatic programmer, operating under a nitrogen (N_2_) inert atmosphere. The furnace temperature was ramped from room temperature to 800 °C, at a rate of 3 °C/min under a continuous flow of N_2_ gas. Upon attaining the desired temperature, the sample was kept at 800 °C for 10 h to finalize carbonization. After carbonization, the nitrogen flow was maintained during cooling until the system returned to room temperature. The resulting material was labeled as CAR_ALD.

CAR_ALD activation was performed in a horizontal furnace with automated temperature regulation. The process began with nitrogen purging until 800 °C was reached. Once the target temperature was attained, the atmosphere was replaced with carbon dioxide (CO_2_) flowing at 50 cm^3^/min, under which the sample was held for 2 h. After activation, nitrogen was reintroduced and maintained during cooling to ambient temperature. The activated carbon obtained was labeled ACT_ALD.

The mixture of RAW_ALD and Bi_2_O_3_-Sm composites was prepared to achieve a final mass ratio of 5:1 following the carbonization process. To obtain an in situ modified material, the mixture was placed in a steel carbonization boat. The rest of the procedure was the same as for the previous materials. The resulting materials are designated as CAR_ALD/Bi_2_O_3_-Sm and ACT_ALD/Bi_2_O_3_-Sm. Data on the mass yield of carbonization and activation are given in Table 1.

#### 2.2.4. Preparation of CPE Working Electrode

From the synthesized materials, eight different carbon pastes were prepared for carbon paste electrodes (CPE) as working electrodes. CPEs were prepared according to the procedure described in Table 2, respecting a solid/liquid ratio of 80%/20%. The components were mixed in a mortar until homogeneous pastes were formed. These pastes were dried for 24 h at room temperature.

### 2.3. Methods

#### 2.3.1. Lignocellulose Composition of Almond Shells

Almond shells were subjected to lignocellulosic composition analysis. The material was ground in a hammer-type micro-mill, and fractions with particle sizes ranging from 0.4 to 1 mm were separated by sieving for chemical analysis. Moisture content of RAW_ALD was determined gravimetrically following TAPPI standard T 264 cm-97 [42]. Cellulose content was quantified using the Kurschner-Hoffer method [43]. For lignin determination, preliminary extraction was carried out in a Soxhlet apparatus with a toluene–ethanol solvent system (2:1, *v*/*v*) to eliminate all aromatic extracts. The second step was to determine Klason’s lignin content, according to the procedure TAPPI T 222 0m-1 [44], in two-stage hydrolysis in sulfuric acid: 72% H_2_SO_4_, for 2 h at room temperature, and then 3% H_2_SO_4_, for 1 h at boiling temperature.

Lignin content of hydrolysate was determined on a UV-VIS spectrophotometer, absorbance at 205 nm (Evolution 300 UV-Vis spectrophotometer, Thermo Electron Corporation, Altrincham, UK) (TAPPI T UM 250 [45]). The content of water-soluble extractives was determined by extraction for 3 h (ASTM D1110-21 [46]). The ash content was determined by complete combustion at 900 °C (TAPPI T 413 om-22 [47]). The content of hemicelluloses is determined approximately, supplementing the content of all components up to 100%.

#### 2.3.2. Nitrogen Physisorption

Textural properties of the RAW_ALD and carbon materials were assessed via N_2_ adsorption–desorption isotherms. These measurements were obtained at a cryogenic temperature of −196 °C using the gas sorption analyzer Sorptomatic 1990 (Thermo Finnigan, Milano, Italy).

Sample preparation, preceding the adsorption analysis, differed for the RAW_ALD and the carbonization obtained materials. The RAW_ALD sample underwent a two-stage degassing protocol: an initial 18 h at room temperature under vacuum, followed by an additional 24 h at 60 C under a vacuum better than 0.3 Pa. The carbon materials were prepared by applying a similar vacuum protocol, but at different temperatures: 2 h at room temperature, and an additional 18 h at 300 C. In both cases, the final step maintained the same residual pressure.

The obtained isotherms were analyzed employing dedicated software, Advanced Data Processing Ver. 5.13 (Thermo Electron, Waltham, MA, USA). The specific surface areas (SSA) were calculated using the Brunauer-Emmet-Teller (BET) equation [48] from a segment of the adsorption isotherms selected according to the Rouquerol criteria [49]. Total pore volume (V_tot_) was determined based on Gurevitsch’s rule, evaluated at a high relative pressure of p/p_0_ = 0.98. The p and p_0_ parameters denote the equilibrium and saturation N_2_ pressures, respectively, at the adsorption temperature. The micropore volume (V_mic_) was determined by applying the Dubinin-Radushkevich method (V_mic-DR_) [50] and the Horvath-Kavazoe method (V_mic-HK_) [51], and the latter was also used to determine the micropore size distribution. The latter was also utilized to assess the micropore size distribution. The Dollimore and Heal method [52], utilizing the adsorption data, was employed to estimate the mesopore volume (V_mes-DH_).

#### 2.3.3. X-Ray Diffraction (XRD) Analysis

The phase composition of the crystalline structure was determined by X-ray diffraction (XRD). Measurements were carried out on a Philips PW-1710/1820 automated diffractometer (Philips, Eindhoven, The Netherlands) with a Cu radiation source (λ = 1.54178 Å), operated at 40 kV and 30 mA. The system was equipped with a curved graphite monochromator and an Xe-filled proportional counter. Diffraction data were collected over a 2θ range of 4–65°, with a step size of 0.02° and a counting time of 1 s per step. Divergence and receiving slits were fixed at 1 and 0.1 units, respectively. Analyses were conducted at room temperature with the sample mounted in a stationary holder.

#### 2.3.4. Fourier Transform Infrared Spectroscopic Analysis (FTIR)

Functional groups in the materials were characterized by diffuse reflectance infrared Fourier transform (DRIFT) spectroscopy. Each sample was thoroughly mixed with anhydrous KBr in a 1:100 (*w*/*w*) proportion using a mortar to obtain a homogeneous blend. The prepared mixture was transferred into a sample cup, and spectra were collected at room temperature using a Perkin Elmer Spectrum Quant spectrometer (PerkinElmer, Inc., Beaconsfield, UK) within the 4000–400 cm^−1^ range.

#### 2.3.5. X-Ray Photoelectron Spectroscopic Analysis (XPS)

XPS analysis of six samples was conducted with a Genesis spectrometer (Physical Electronics Inc., Chanhassen, MN, USA) equipped with an Al monochromatic source. The spectra provided information on elemental surface composition and chemical bonding states. The probed area was 500 μm in diameter, with a sampling depth of 3–5 nm. Photoelectron spectra were recorded with an energy resolution of 0.65 eV. Two regions of each sample were analyzed, and the spectra showed excellent reproducibility. The binding energy uncertainty was ±0.4 eV. Quantification of surface composition was based on peak intensities, corrected using relative sensitivity factors supplied by the instrument manufacturer [53]. Data were processed with Multipak software (version 9.9).

#### 2.3.6. Thermogravimetric and Differential Thermal Analysis (TG-DTA)

The investigated samples were simultaneously analyzed by non-isothermal thermogravimetric analysis (TGA) and differential thermal analysis (DTA) using a Setaram Setsys Evolution 1750 system (Setaram, Lyon, France). Experiments were conducted in an inert atmosphere with a controlled gas flow of 10 cm^3^/min.

#### 2.3.7. Electrochemical Characterization

Electrochemical characterization was performed using a PalmSens4 potentiostat with PS Trace 5.8 software (PalmSens BV, Houten, The Netherlands). A three-electrode cell was employed, consisting of Ag/AgCl (3 M KCl) as the reference electrode, a platinum wire as the counter electrode, and a carbon paste electrode incorporating the synthesized material as the working electrode. EIS measurements were conducted in a 5 mM equimolar mixture of K_3_[Fe(CN)_6_] and K_4_[Fe(CN)_6_] in 0.1 M KCl, across a frequency range of 0.01 Hz–100 kHz, with a perturbation amplitude of 10 mV under static conditions. Cyclic voltammetry was performed in the same Fe^2+^/Fe^3+^ electrolyte, within a potential range of −0.5 to 1.0 V, at a scan rate of 50 mV/s.

## 3. Results and Discussion

### 3.1. Lingocellulose Composition

Insight into the lignocellulose composition can predict which type of carbon will be obtained after pyrolysis. The RAW_ALD used in this study is rich in lignocellulosic matrix, with a moisture content of 8.44%, ash 0.54%, and extractives 3.64%. The complete description of the lignocellulose content is presented in Table 3.

Based on the results presented in Table 3, the cellulose and hemicelluloses contents were found to be 34.25% and 13.48%, respectively. The total polysaccharide content in the analyzed almond shells does not deviate significantly from the values reported in the literature, where cellulose typically ranges from 25–35%, and hemicelluloses from 18–30% [9,54]. As the most crystalline component of the lignocellulosic matrix, cellulose contributes to the thermal and mechanical stability of the material, whereas hemicelluloses, due to their amorphous nature, play a key role in pore formation during carbonization [55].

In contrast, the lignin content in the sample is up to 20% higher than the commonly reported values for almond shells, which typically range from 28–35% [9,54]. This variation can be attributed to a combination of factors, including almond variety, regional growing conditions, climate, and ripeness of the fruit at harvest.

A higher lignin content can be advantageous for applications requiring improved mechanical integrity and chemical resistance. Lignin is a highly branched polyaromatic macromolecule that forms covalent bonds with cellulose and hemicelluloses, thereby reinforcing the rigidity of the plant structure [56]. Its aromatic nature plays a critical role in the formation of conjugated carbon networks during pyrolysis, which is essential for producing functional carbon materials [57]. Additionally, the hydrophobic nature of lignin enhances the moisture and chemical resistance of lignin-based composites, making it especially valuable for durable biocomposites and structural materials [58].

### 3.2. Nitrogen Physisorption

**Figure 1:**
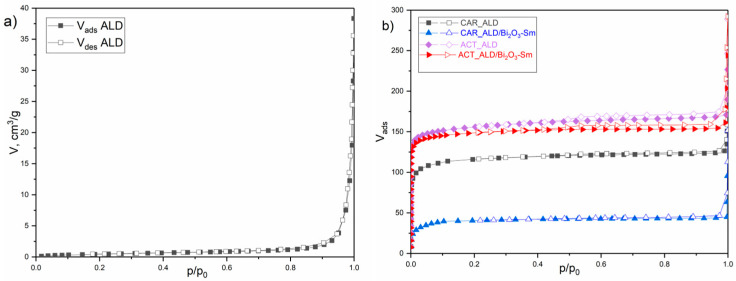
Nitrogen adsorption–desorption isotherms of: (**a**) RAW_ALD and (**b**) carbonized and activated materials.

Nitrogen physisorption measurements investigated the textural properties of almond shells and synthesized carbon at −196°C. The corresponding N_2_ adsorption/desorption isotherms are presented in Figure 1, and the calculated textural properties are summarized in Table 4.

The N_2_ adsorption isotherm of the RAW_ALD sample is convex to the p/p_0_ axis over the entire relative pressure range, does not exhibit an inflection point within the BET region, and shows a small total adsorbed volume. Its shape is most similar to Type III according to the IUPAC classification, which is characteristic of non-porous materials with weak adsorbent-adsorbate interactions [59].

Consistent with this isotherm type, the RAW_ALD sample possesses a very low specific surface area (SSA_BET_) of approximately 2 m^2^/g and a C_BET_ constant of about 15. Such a low C_BET_ value precludes a reliable determination of the monolayer capacity and, consequently, an accurate SSA_BET_ calculation.

Furthermore, as the models for quantifying micro- and mesoporosity are unsuitable for this isotherm type, only the total pore volume (V_tot_) is reported for RAW_ALD in Table 4. Thus, the SSA_BET_ value should be interpreted only qualitatively, and not as the true specific surface area.

The isotherms for all four carbon materials differ significantly from the RAW_ALD isotherm and exhibit a distinctive shape typical of many carbon materials presented in the literature [49]. Isotherms of this shape are most often classified as Type Ib according to the IUPAC nomenclature. Indeed, a sharp uptake at low relative pressures (p/p_0_ ≤ 0.01), a transition segment with a moderate increase up to p/p_0_ ≈ 0.2 (corresponding to supermicropores and small mesopores), followed by a long, nearly horizontal plateau across the largest p/p_0_ region, and a final increase in volume near p/p_0_ = 1 is characteristic of this isotherm type.

In addition to differences in relation to the raw almond isotherm, the different synthesis processes applied result in mutual differences both through isotherms and through differences in the measured textural properties of the resulting carbonized materials (Table 1).

For example, carbonization increased the total pore volume (V_tot_) of CAR_ALD by almost 13 times and the specific surface area by over two orders of magnitude compared to RAW_ALD, highlighting the efficiency of the process in terms of developing textural properties.

Subsequent CO_2_ activation procedure further enhanced the textural properties of ACT_ALD, increasing the SSA_BET_ by ~19% and V_tot_ by ~35% relative to CAR_ALD. Comparable to the V_tot_ increase, the micropore volumes calculated by the Horvath-Kawazoe and Dubinin-Radushkevich methods showed a similar percentage increase of 36% and 32%, respectively. Although the absolute mesopore volume remained low (~0.034 cm^3^/g), it exhibited the largest relative increase at ~55%.

The observed difference in the percentage increase across the different pore classes suggests a non-uniform influence of the CO_2_ activation process. Evidently, the CO_2_ primarily affects the micropores and, to some extent, the smallest mesopores. The mechanism of the physical activation of carbonized materials is well-researched and involves preferential targeting of energetically unstable sites [60]. The action of the activator molecules on these preferential sites leads to a significant increase in both the micropore volume and the relative proportion of mesopores, compared to the non-activated sample. The barely noticeable hysteresis loop of the CAR_ALD sample becomes more pronounced after CO_2_ activation. The shape of these loops is unequivocally of the H4 type, which is characteristic of micro-mesoporous carbons [59] and indicates that the adsorption–desorption isotherms are not fully reversible [49]. The textural properties of the CAR_ALD/Bi_2_O_3_-Sm and ACT_ALD/Bi_2_O_3_-Sm samples differ significantly from those of their unmodified counterparts, yet in distinctly different manners. The CAR_ALD sample experienced a drastic loss of textural properties, losing approximately 64% of its specific surface area and total pore volume following the Bi_2_O_3_-Sm modification (e.g., SSA_BET_ dropped from 451 m^2^/g to 163 m^2^/g). This massive reduction, accompanied by a reduction in micropore volume also by ≈64%, alongside a doubling of the maximum pore width (D_max-HK_) from 0.53 nm to 1.09 nm, suggests pore blocking—likely by Bi_2_O_3_-Sm nanoparticles occluding narrow micropore entrances and rendering most of the internal surface inaccessible.

Conversely, the CO_2_-activated sample with modifier (ACT_ALD/Bi_2_O_3_-Sm) showed only a negligible loss of SSA_BET_ (≈1.3% loss, 535 m^2^/g vs. 528 m^2^/g) and 4.2% reduction in micropore volume compared to its non-modified counterpart (ACT_ALD). This finding is particularly significant considering that the carbonaceous phase—de facto, the sole contributor to the surface area—constitutes only 76.05% of the mass in the modified sample (due to the 23.95% modifier content), whereas ACT_ALD is completely carbonaceous material. The modifier (Bi_2_O_3_-Sm) promotes the catalytic degradation of the carbon scaffold during the CO_2_ activation process, thereby simultaneously opening previously blocked pores and creating new ones. This leads to the development of a more complex porous structure despite the lower relative amount of the carbon phase. If the blocking mechanism were the dominant cause of the manifested textural properties here as well, their even more drastic decline would be expected, which is not the case.

### 3.3. X-Ray Diffraction Analysis

**Figure 2:**
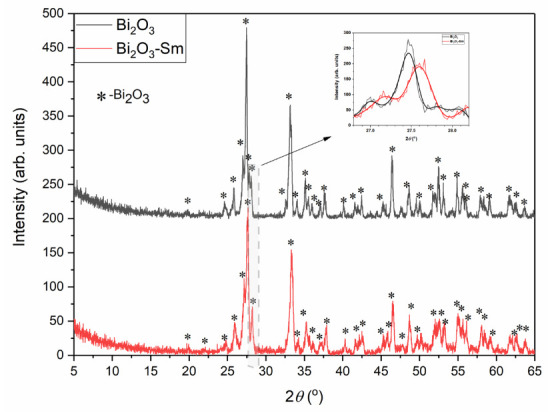
XRD patterns of Bi_2_O_3_ and Bi_2_O_3_-Sm composite.

The crystallographic structures of all materials were identified using X-ray diffraction. As shown in Figure 2, the analyzed Bi_2_O_3_ material (black curve) consists exclusively of Bi_2_O_3_ (ICDD 01-071-0465). The crystallographic structure of the Bi_2_O_3_-Sm material (red curve) reveals the Bi_2_O_3_ phase, but the diffraction peaks are shifted towards higher 2θ angles, indicating smaller unit cell parameters [61]. This shift is attributed to doping with Sm (5%), where smaller Sm ions substitute larger Bi ions. A noticeable shift is observed from 27.46° for Bi_2_O_3_ to 27.60° for Bi_2_O_3_-Sm, as illustrated by the red curve in the second graph of Figure 2.

**Figure 3:**
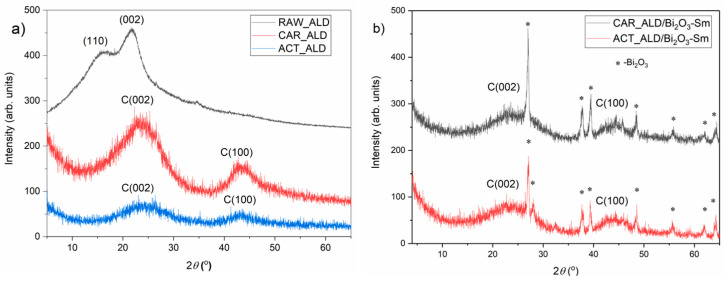
XRD patterns of (**a**) raw, carbonized and activated samples before Bi_2_O_3_-Sm modification, and (**b**) carbonized and activated samples after Bi_2_O_3_-Sm modification.

The XRD pattern of RAW_ALD is presented in Figure 3a. As shown, two diffraction peaks are observed at 2θ = 16.6° and 2θ = 21.8°, corresponding to the planes of (110) and (002), which are attributed to crystalline regions of cellulose type I [62]. A low-intensity peak appearing around 34.6° may originate from mineral components such as calcium carbonate, silicates, or other inorganic phases. The results obtained in this study agree with the results reported by Li et al. [9].

Li et al. [9] also investigated the structural characteristics of almond shells and attributed Li et al. also investigated the structural characteristics of almond shells and attributed the peak near 2θ ≈ 22.6° to the (002) crystallographic plane. Additional peaks at approximately 14.8° and 16.4° were associated with the (101) and (100) planes, respectively. Furthermore, a broad background signal between 18° and 21° was interpreted by the authors as an indication of amorphous regions.

The X-ray diffraction curves for CAR_ALD and ACT_ALD each exhibit two broad peaks, located near 23.5° and 43.5° [63]. The first peak corresponds to the crystal plane index C (002), associated with the parallel and azimuthal orientation of the aromatic and carbonized structure. The second broad peak, observed at 43.5°, is assigned to C (100) diffractions of graphitic and hexagonal carbons, reflecting the size and arrangement of the aromatic laminae [64]. The decrease in peak intensity for ACT_ALD compared to CAR_ALD suggests that activation reduces the already limited structural ordering domains, as reflected by the (002) and (100) planes.

Figure 3b presents the XRD patterns of carbon and activated materials modified with Bi_2_O_3_-Sm composites (mass ratio 5:1). The analyzed samples represent a mixture of CAR_ALD and Bi_2_O_3_-Sm (black line) and activated ALD and Bi_2_O_3_-Sm (red line), which are predominantly amorphous structures with two broad peaks characteristic for carbon structure, but also show sharp peaks, which confirms presence of the modifier (Bi_2_O_3_-Sm).

### 3.4. Fourier Transform Infrared Spectroscopic Analysis (FTIR)

**Figure 4:**
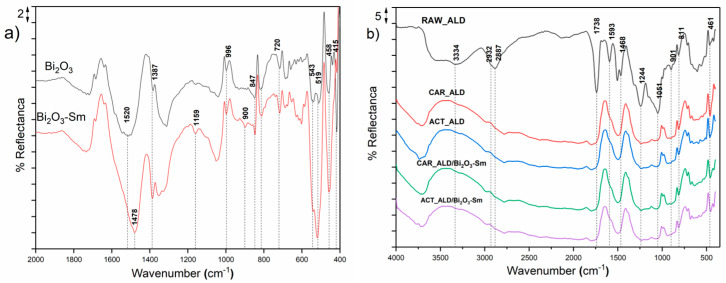
DRIFT spectra of (**a**) Bi_2_O_3_ and Bi_2_O_3_-Sm materials, (**b**) raw and carbon materials.

To investigate the structural properties of the synthesized oxides and how the carbonization process affects the structure of the waste almond shells, FTIR spectra were recorded using the DRIFT technique. The recorded spectra are shown in Figure 4.

The DRIFT spectra of synthesized Bi_2_O_3_ (black curve) and Bi_2_O_3_-Sm (red curve), as shown in Figure 4a, reveal similar and distinct vibrational bands that provide insights into the structural modifications induced by Sm doping. In the spectrum of Bi_2_O_3_, the following spectral bands are visible: 415, 458, 519, 543, 720, 847, 996, 1387, and 1520 cm^−1^. The spectral bands in the range of 415–543 cm^−1^ originate from the stretching vibrations of Bi–O bonds in [BiO_6_]^9−^ octahedral units. The spectral bands at 720 and 847 cm^−1^ originate from the symmetric stretching vibrations of Bi–O bonds in [BiO_3_]^3−^ pyramidal units [65]. Stretching vibrations of Bi–O bonds also contribute to the spectral bands at 996 and 1387 cm^−1^ [66,67]. The band at 1520 cm^−1^ originates from the N=O bending vibrations that lag during synthesis [68,69]. Doping Bi_2_O_3_ with Sm^3+^ ions induce structural modifications at the atomic level, affecting Bi–O bond lengths and altering vibrational frequencies, as evidenced in the FTIR spectra. The transformation of bridging oxygens (BOs) into non-bridging oxygens (NBOs) alter the connectivity within the oxide network, often leading to shifts in peak positions and variations in intensity [70].

This change not only affects the phonon modes of the material but also plays a crucial role in modifying its optical, electrical, and mechanical properties. The presence of Sm can introduce localized distortions, influencing factors like bandgap, density, and even ionic conductivity, making it useful for applications in photonic devices and radiation shielding materials [71,72].

As shown in Figure 4b, the DRIFT spectrum of the RAW_ALD material reveals a diverse array of functional groups, indicating its complex composition. The RAW_ALD material, being a biological material, predominantly consists of lignin, cellulose, and hemicellulose. Additionally, XRD analysis of the carbonized materials identified a graphite structure.

The spectrum of the RAW_ALD material shows the characteristic stretching vibrations of lignocellulose. The broad peak at 3334 cm^−1^ indicates the stretching vibration of O–H groups from cellulose and hemicelluloses [73]. The doublet at 2887 and 2932 cm^−1^ originates from the stretching vibrations of methyl and methylene (C–H) groups. The band at 1744 cm^−1^, corresponding to the stretching vibrations of C=O groups, indicates the presence of hemicellulose, along with the peak at 1051 cm^−1^, which originates from C–O stretching vibrations. The presence of lignin is evident based on stretching vibrations at 1593 cm^−1^ (benzene ring), 1468 cm^−1^ (–CH_3_O), and 1245 cm^−1^ (C–C, C–O). Additional peaks arise from the bending vibration at 901 cm^−1^ of R_2_C=CH_2_, while the band at 811 cm^−1^ represents overtones of the benzene structure [9].

The carbonization and activation processes (CAR_ALD and ACT_ALD samples) result in the loss of peaks characteristic of cellulose and hemicellulose, leaving only the peak characteristic of lignin at 1468 cm^−1^, which is slightly shifted to 1489 cm^−1^ and the band at 811 cm^−1^ (overtones of the benzene structure). This change indicates the complete degradation of the cellulosic components and partial degradation of the lignin-based composition. However, after carbonization, a peak confirming the formation of graphite appears at 461 cm^−1^ [74].

The formation of the graphite can be explained as follows. The carbonization of cellulose and hemicelluloses starts at a temperature of 350 °C, splitting the glycosidic bonds. Increasing the temperature leads to decarbonization of these compounds to form polyaromatic compounds, resulting in carbon. This process in cellulose occurs through the formation of levoglucosan, but hemicelluloses do not form levoglucosan due to the absence of the six-carbon and substituted oxygen at position four [75,76]. Regarding lignin, it is stable up to 400 °C. Above this temperature, G-lignin and S-lignin are transformed into polycyclic aromatic hydrocarbons. Increasing the temperature to 800 °C, dehydrogenation converts these intermediates into carbon [76,77]. The recombination of carbonization products can lead to the formation of graphite, which was observed by XRD analysis.

In the spectra of the CAR_ALD/Bi_2_O_3_-Sm and ACT_ALD/Bi_2_O_3_-Sm samples, no new spectral bands indicating the formation of new phases or significant structural changes were detected. Therefore, based on the FTIR results, it can be concluded that modification did not affect the structural properties of the samples, as expected, since only physical mixing was used for their preparation. Additionally, no characteristic spectral bands for Bi_2_O_3_ or Sm were observed, likely due to the very low amounts used for modification (less than 5%).

### 3.5. X-Ray Photoelectron Spectroscopic Analysis (XPS)

**Figure 5:**
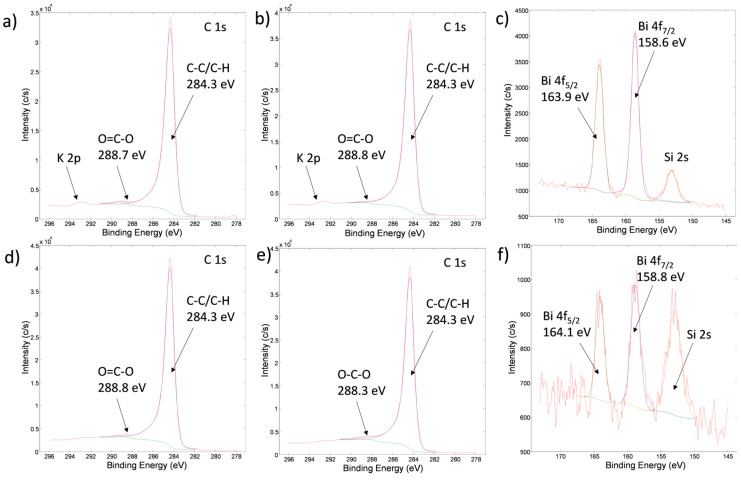
High-energy resolution XPS spectra C 1s and Bi 4f from surfaces of samples: (**a**) C 1s from CAR_ALD, (**b**) C 1s from CAR-ALD/Bi_2_O_3_-Sm, (**c**) Bi 4f from CAR-ALD/Bi_2_O_3_-Sm, (**d**) C 1s from ACT_ALD, (**e**) C 1s from ACT-ALD/Bi_2_O_3_-Sm, (**f**) Bi 4f from ACT-ALD/Bi_2_O_3_-Sm.

High-energy resolution XPS spectra of C 1s and Bi 4f were acquired on carbon materials to identify chemical bonds of elements on the surface. The spectra are shown in Figure 5.

The carbon C 1s spectra (Figure 5a,b,d,e) from carbon materials are similar. They were deconvoluted into two peaks. The main peak (99% of the total spectrum) for all samples is at 284.3 eV, and it is related to C-C/C-H bonds. Another feature of this peak is an asymmetric tail on the high binding energy side around 286 eV. This is very specific for graphitic-like bonded carbon atoms. This shows that after carbonization and activated treatment, the majority of C-atoms on the surface are in the graphitic-like phase. This also confirms the FTIR results. In addition to the graphitic-like features, there are also small peaks (1% of the total spectrum) in the C 1s spectra at around 288.8 eV, which are related to O=C-O bonds. This peak reflects the presence of a small concentration of carboxylic-like surface functional groups on the graphitic matrix. For CAR_ALD and CAR_ALD/Bi_2_O_3_-Sm (Figure 5a,b), at an energy of 292.5 eV, small peaks can also be identified, which are assigned to K 2p spectra. They are present to a larger extent on these materials, which were not activated. The concentration of K for these two samples is about 0.3–0.5 at.%.

**Figure 6:**
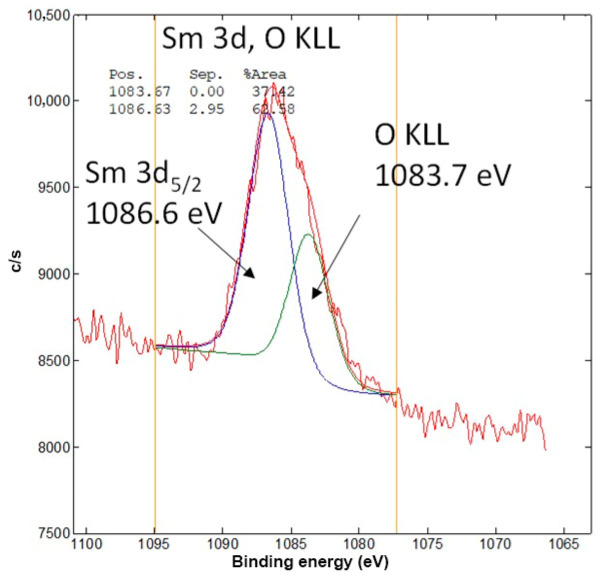
High-energy resolution XPS spectrum of: Sm 3d5/2, from the surface of the sample Bi_2_O_3_-Sm.

Bi 4f spectra were detected on CAR_ALD/Bi_2_O_3_-Sm and ACT_ALD/Bi_2_O_3_-Sm (Figure 5c,f). They consist of a Bi 4f7/2 peak at approximately 158.7 eV and a Bi 4f5/2 peak at approximately 164.0 eV. Both of them are related to the oxidation state Bi^3+^ in the Bi_2_O_3_-like species [37]. The average surface concentration of Bi is about 0.15 at.% for CAR_ALD/Bi_2_O_3_-Sm and 0.05 at.% for ACT_ALD/Bi_2_O_3_-Sm. The Sm 3d5/2 peak at an energy of 1086.2 eV (Figure 6) was not detected on the surfaces of CAR_ALD/Bi_2_O_3_-Sm and ACT_ALD/Bi_2_O_3_-Sm samples, due to low concentration.

### 3.6. Thermogravimetric and Differential Thermal Analyses (TGA-DTA)

**Figure 7:**
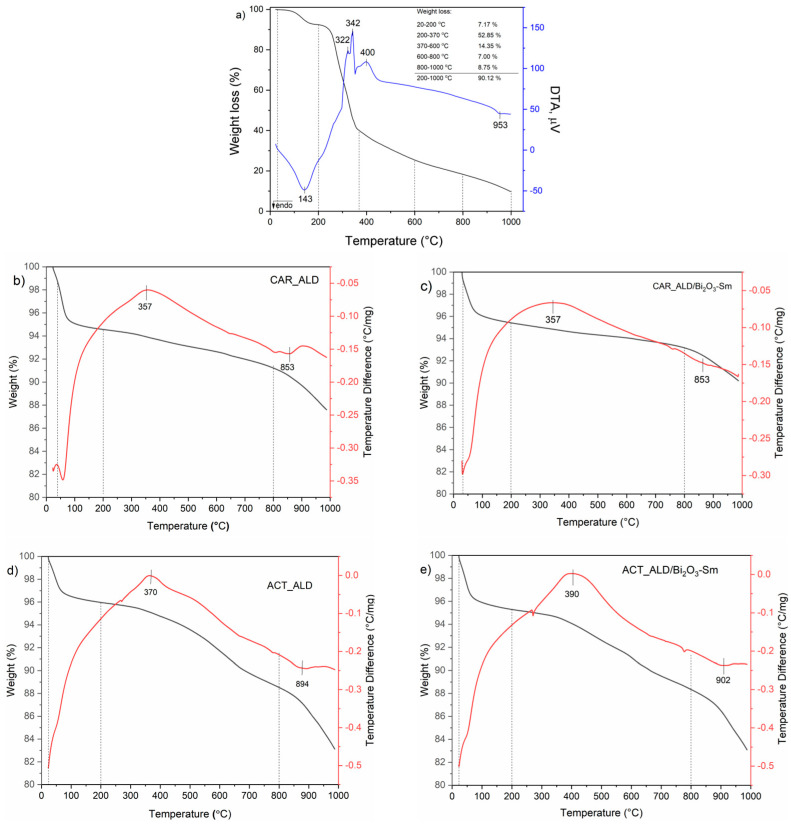
TGA-DTA curves of: (**a**) RAW_ALD, (**b**) CAR_ALD, (**c**) CAR_ALD/Bi_2_O_3_-Sm, (**d**) ACT_ALD, (**e**) ACT_ALD/Bi_2_O_3_-Sm.

Results of thermal analyses of the raw almond shells sample, as well as carbonized and activated samples, unmodified and modified, are presented in Figure 7.

As can be seen from Figure 7a in the DTA diagram of the RAW_ALD, four peaks are visible, at 143, 322, 342, 400 and 953 °C. Also, thermal decomposition can be divided into five temperature intervals: 25–200; 200–375, 375–600, 600–800 and 800–1000 °C. In the first temperature interval (25–200 °C), the endothermal DTA peak at 143 °C can be assigned to the water (moisture) release and is followed by 7.17% weight loss. In the second temperature interval (200–375 °C) the main weight loss occurs (52.85%) and is a consequence of the decomposition of the main organic compounds, i.e., devolatilization/decomposition of hemicelluloses and cellulose, which is followed by the exothermal DTA peaks at 322 and 342 °C, respectively. Lignin is the more thermostable component whose decomposition takes place slowly over a greater temperature range (375–800 °C) and is followed by the exothermal peak at 400 °C [55]. Dehydrogenation converts intermediates’ decompositions into carbon by increasing the temperature from 600 to 800 °C. The recombination of carbonization products leads to the formation of graphite, which can further be oxidized to CO and CO_2_. However, as the thermal analysis was conducted in an inert atmosphere, graphite oxidation was not possible. Thus, total mass loss in the range of 370–800 °C results solely from the thermal degradation of lignite. Thermal analysis confirms the presence of graphite across the endothermal DTA peak at 953 °C, which can be associated with mild endothermic changes related to the structural rearrangement of graphite or possible sublimation at temperatures close to 1000 °C [78].

The TGA-DTA diagrams of the CAR_ALD can be divided into three temperature intervals: 25–200 °C, 200–800 °C, and 800–1000 °C. After carbonization, the DTA peaks characteristic of hemicelluloses and cellulose decomposition disappear, and the subsequent TGA weight loss is negligible (0.71% in the 200–375 °C interval), which is a consequence of their complete decomposition during the carbonization process. The total weight loss in the 25–1000 °C range was 11.44%, of which 4.16% resulted from moisture (water) release (25–200 °C). The very low weight loss (3.15%) in the 200–800 °C interval is primarily due to the additional decomposition of lignin residues after the carbonization process. This is accompanied by an exothermic DTA peak, initially observed at 400 °C, which shifts to 357 °C. This shift towards lower temperatures is expected, given that the sample undergoes thermal treatment after already being thermally treated during the carbonization process. As a result, the secondary decomposition of residual lignin occurs more easily compared to the initial raw material. The weight loss of 4.13% in the last temperature interval (800–1000 °C) can also be associated with mild endothermic changes, related to the additional structural rearrangement of graphite or sublimation at temperatures close to 1000 °C. This is also accompanied by a DTA peak at 953 °C, which shifts to a lower temperature (853 °C), which can be explained in the same way as for the peak at 400 °C. The addition of Bi_2_O_3_-Sm in CAR_ALD/Bi_2_O_3_-Sm did not cause significant changes in its thermal properties. The DTA diagrams for CAR_ALD/Bi_2_O_3_-Sm remained nearly identical, and the weight losses in TGA diagrams across specific temperature intervals, as well as the total weight loss, did not differ significantly (~1%) along with the DTA peaks.

The results of the TGA-DTA analyses of the ACT_ALD are also present in Figure 7d. As can be seen, the TGA diagram can be divided in the same way as for the carbonized sample. In the first temperature interval (25–200 °C) the weight loss from the release of moisture (water) was 3.63%. In the second temperature interval (200–800 °C) the weight loss on TGA diagram can be explained by the thermal decomposition of thermally unstable groups remaining in the sample after the carbonization process, specifically those originating from hemicelluloses and cellulose (in the temperature range of 200–375 °C, with a mass loss of 0.99%), as well as those derived from lignin, which is accompanied by an exothermic peak at 375 °C on the DTA curve. Besides that, activation of the sample with CO_2_ leads to the formation of certain functional groups on the surface of the carbonaceous material, such as carbonyl, carboxyl, and phenolic groups. When heated in an inert atmosphere in the temperature range of 400–800 °C, these groups can be gradually removed through thermal desorption, which means that these functional groups can be relatively unstable and leave the sample surface as gaseous products, such as CO_2_, CO, or H_2_O. Then, in this temperature interval, bond decomposition can also occur and the bonds between carbon and oxygen can break, potentially reducing the oxygen content in the material and altering its surface chemistry. All these processes are responsible for the weight loss of 6.45% in the temperature interval of 375–800 °C. In the temperature interval of 800–1000 °C, significant changes occur in the structure of the carbonaceous material due to reorganization of carbon layers, i.e., carbon atoms gradually rearrange into graphite structures, increasing the crystallinity of the material. Then, these temperatures lead to stabilization and can cause expansion of the porous structure formed during the activation process due to the removal of residual chemical compounds and thermal stability. All of these are followed by the weight loss of 5.43% on the TGA curve, while the presence of the graphite was confirmed by the endothermic DTA peak at 876 °C. In the whole 25–1000 °C temperature interval, total weight loss was 17.49% [79].

The TGA-DTA diagrams of the ACT_ALD/Bi_2_O_3_-Sm sample are shown in Figure 7e. As observed in the case of CAR_ALD/Bi_2_O_3_-Sm, the modifier does not significantly alter the thermal behavior of the activated sample. The overall trends of the DTA and TGA curves remain consistent. Weight losses within specific and total temperature intervals differ only marginally (less than 1%), and the DTA peaks are slightly shifted toward higher temperatures due to the presence of Bi_2_O_3_-Sm. These results indicate that the addition of Bi_2_O_3_-Sm does not substantially affect the thermal properties of the activated sample.

### 3.7. Electrochemical Characterization

**Figure 8:**
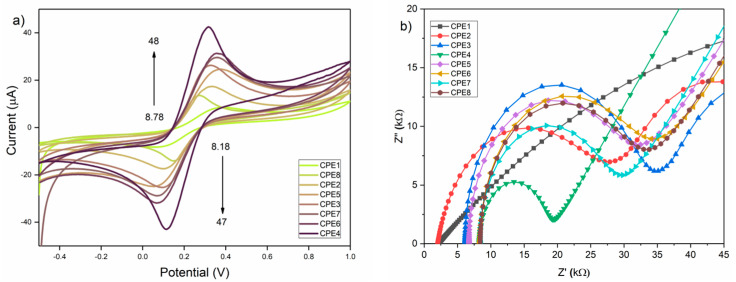
(**a**) CVs of carbon paste materials modified with Bi_2_O_3_ and Bi_2_O_3_-Sm recorded in 0.1 M KCl solution containing 5 mM Fe^2+/3+^, scan rate 50 mV/s; (**b**) EIS responses of the same electrodes in 0.1 M KCl solution containing 5 mM Fe^2+/3+^.

High electrical conductivity represents a fundamental requirement for the development of electrochemical sensors, as it directly influences the efficiency of electron transfer at the electrode–electrolyte interface. The electrochemical performance of the electrodes fabricated from the synthesized materials was evaluated by cyclic voltammetry (CV) and electrochemical impedance spectroscopy (EIS), using a 5 mM Fe^2+^/Fe^3+^ redox couple in 0.1 M potassium chloride as the supporting electrolyte. The results are presented in Figure 8. These techniques provide valuable insights into the redox behavior, charge transfer kinetics, and interfacial properties of the electrode materials, important parameters in assessing their suitability for sensor applications.

As described in the experimental section, eight different carbon paste electrodes (CPEs) were prepared. CPE1 and CPE5 were formed using CAR_ALD and ACT_ALD, respectively. CPE2, CPE3, CPE6, and CPE7 were prepared by manually incorporating Bi_2_O_3_ and Bi_2_O_3_-Sm into carbon pastes, while CPE4 and CPE8 represent in situ modified materials, i.e., those modified with Bi_2_O_3_-Sm before the carbonization process. As shown in Figure 8a, all eight electrodes exhibit defined cathodic and anodic peaks, characteristic of a redox system. Modifying the electrodes with Bi_2_O_3_ and Bi_2_O_3_-Sm enhances the redox peaks more intensely and better distinguishes, leading to an increase in peak currents and a significant decrease in peak-to-peak separation (∆E_p_). Minor shifts in the peak potentials—specifically, the anodic peak moving toward more positive and the cathode peak toward more negative values—are likely indicative of slight surface heterogeneity at the electrode interface. Such deviations are commonly observed in electrodes fabricated under laboratory conditions [80]. An increase in the anodic and cathodic currents is presented in Table 5.

**Figure 9:**
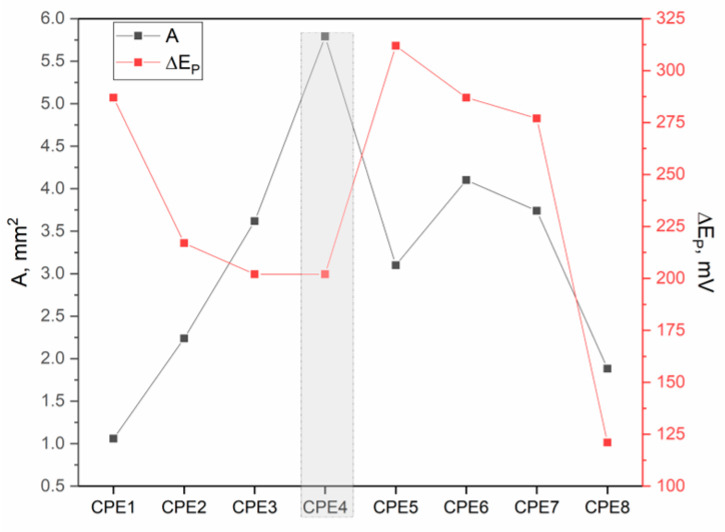
Correlation of electrode performance indicators with electrochemical parameters: electroactive surface area (A)—black scatter pots, and peak-to-peak separation (∆E_p_)—red scatter plots.

There is noticeable increase in the anodic current (I_a_) form 8.78 ± 0.31 µA (CPE1) to 48 ± 1.68 µA (CPE4), and in the cathodic current from 8.18 ± 0.29 µA (CPE1) to 47 ± 0.91 µA (CPE4). This improvement of 546.70% for the anodic and 575.28% for the cathodic current (CPE1 vs. CPE4) is attributed to the presence of modifiers, which improve the electronic environment at the electrode surface by promoting faster electron transfer, increasing conductivity, and exhibiting favorable electrocatalytic activity. These effects are supported by the calculated electroactive surface area values, using the Randles–Sevcik equation:

I_p_ = 2.69 × 10^5^ × n^(3/2)^ × A × D^(1/2)^ × c_0_ × ϑ^(1/2)^ (1)

where n is the number of electrons in the redox system, A is the electroactive surface area (cm^2^), D is the diffusion coefficient of the molecule in solution (cm^2^/s), c_0_ is the concentration of the molecule in solution (mol/cm^3^), and υ is the scan rate (V/s). According to the results in Figure 9, it is observed that CPE4 developed the largest electroactive surface area of 0.058 cm^2^, which confirms the higher availability of active sites. Although the CAR_ALD sample exhibited a reduction in specific surface area following Bi_2_O_3_-Sm modification (~163 m^2^/g), this modification was accompanied by a doubling of the maximum pore width (D_max-HK_), reaching 1.09 nm. The penetration of Bi_2_O_3_-Sm nanoparticles and the partial blockage of narrow micropore entrances enabled greater activation of internal surface functional groups. This interaction between carbonized carbon and Bi_2_O_3_-Sm led to an increase in the number of active sites, resulting in an enhanced electroactive surface. Supporting this, the calculated electroactive surface area reached 0.058 cm^2^. In addition, the ∆E_p_ value has the lowest value for this electrode of 202 mV, which confirms the improved charge transfer kinetics through the electrode. A more detailed examination of Figure 9 reveals a consistent trend in both the electroactive surface area and ∆E_p_ values. Specifically, ∆E_p_ decreases gradually from CPE1 to CPE4, and from CPE5 to CPE8, which can be attributed to the introduction of Bi_2_O_3_ and Bi_2_O_3_-Sm modifiers that enhance electron-transfer kinetics. Similarly, the electroactive surface area increases from CPE1 to CPE4, and from CPE5 to CPE7, as expected. However, CPE8 shows a pronounced decline in the surface area, accompanied by a marked reduction in anodic and cathodic peak currents. This phenomenon is likely due to the modifier penetrating and becoming entrapped within the microporous structure of the carbon during activation, rendering a portion of the surface electrochemically inactive. As a result, these regions do not effectively participate in the electrochemical reaction, leading to a reduced overall electroactive area.

Electrochemical impedance spectroscopy (EIS) was conducted in the same test solution to confirm these findings further, and the corresponding Nyquist plots are presented in Figure 8b). Equivalent circuit modeling revealed a significant decrease in charge transfer resistance (R_ct_) from CPE1 > CPE3 > CPE6 > CPE2 > CPE5 > CPE8 > CPE7 > CPE4 (11.18 kΩ), as shown in Table 5. This indicates enhanced electron transfer at the electrode-electrolyte interface, as demonstrated by CV (Figure 8a). Additionally, linearity in the low-frequency field was observed for this electrode, indicating the diffusional movement of ions within the electrochemical system. These results reinforce the beneficial role of the synthesized materials in lowering interfacial resistance and promoting efficient charge transport.

Based on the combined evaluation of peak current intensity, peak-to-peak separation, and electroactive surface area, CPE4 (CAR_ALD/Bi_2_O_3_-Sm) exhibits the best electrochemical performance and is considered the most efficient electrode in this study. The conclusion is further supported by EIS, which confirms its superior charge transfer properties. As shown in Table 6, the proposed CPE4 electrode fabricated from CAR_ALD/Bi_2_O_3_-Sm displays electrochemical properties that are comparable to or superior to those reported in the literature.

The results of this study highlight the potential of agricultural residues for use in advanced sensor technologies and offer a valuable contribution to the broader fields of green chemistry and sustainable materials science.

## 4. Conclusions

This study successfully demonstrated the valorization of almond shell waste as a sustainable carbon source for fabricating carbon paste electrodes (CPEs). The lignocellulosic composition of the raw material—34.25% cellulose, 13.48% hemicellulose, and 48.03% lignin provided a robust foundation for carbon development. Carbonization significantly enhanced the textural properties of CAR_ALD, increasing its total pore volume nearly 13-fold and its specific surface area by more than two orders of magnitude compared to RAW_ALD. Subsequent CO_2_ activation further improved ACT_ALD, yielding a ~19% increase in surface area and ~35% increase in pore volume relative to CAR_ALD. Physico-chemical analyses confirmed the successful completion of both carbonization and activation processes. The incorporation of Bi_2_O_3_-Sm as a surface modifier further improved the electrochemical performance of the biocarbon. XRD and FTIR analyses revealed the formation of new C–C bonds indicative of graphitic structures, while XPS confirmed the presence of Bi_2_O_3_-Sm on the carbon surface. Thermal analysis showed that the addition of Bi_2_O_3_-Sm did not significantly alter the thermal stability of either the carbonized or activated samples.

Electrochemical testing revealed the highest current response (48 µA) for the CPE fabricated from CAR_ALD/Bi_2_O_3_-Sm, underscoring the synergistic effect of surface modification. Notably, this work reports, for the first time, the successful fabrication of a fully functional CPE working electrode derived entirely from agricultural waste. These findings highlight the potential of low-cost, biowaste-derived carbon, enhanced with Bi_2_O_3_-Sm, as a sustainable and high-performance platform for electrochemical applications. Future research will focus on optimizing material functionality and evaluating sensing performance in realistic environments to unlock the full potential and amplify the impact of this eco-friendly technology.

## Figures and Tables

**Table 1:** The mass yields of carbon materials.

Mass	CAR_ALD	CAR_ALD/Bi_2_O_3_-Sm	ACT_ALD	ACT_ALD/Bi_2_O_3_-Sm
m_0_ (g)	42.16	42.85	11.00	8.01
m_f_ (g)	11.82	13.40	9.82	6.24
ω (%)	28.03	31.27	89.27	77.90

m_0_—mass of material before carbonization/activation process; m_f_—mass of material after carbonization/activation process; ω (%)—mass yields of carbonization/activation.

**Table 2:** CPEs preparation process.

Mass%	CAR_ALD	CAR_ALD/Bi_2_O_3_-Sm	ACT_ALD	ACT_ALD/Bi_2_O_3_-Sm	Modifiers		Paraffine Oil
					Bi_2_O_3_	Bi_2_O_3_-Sm	
CPE1	80						20
CPE2	66.6				13.4		20
CPE3	66.6					13.4	20
CPE4		80					20
CPE5			80				20
CPE6			66.6		13.4		20
CPE7			66.6			13.4	20
CPE8				80			20

**Table 3:** Lignocellulose content of almond shells.

	Cellulose, %	Hemicelluloses, %	Lignin, %
RAW_ALD [this study]	34.25	13.48	48.09
Almond shell [54]	34.39	13.96	39.92
Almond shell [9]	38.47	28.82	29.54

**Table 4:** Textural properties of RAW_ALD, carbonized and activated materials before and after modification with Bi_2_O_3_-Sm.

	RAW_ALD	CAR_ALD	CAR_ALD/Bi_2_O_3_-Sm	ACT_ALD	ACT_ALD/Bi_2_O_3_-Sm
SSA_BET_, m^2^g^−1^	2	451	163	535	528
C_BET_	15	1240	159	58,270	42,790
V_tot_, cm^3^g^−1^	0.015	0.194	0.069	0.262	0.244
V_mes-DH_, cm^3^g^−1^	-	0.022	0.009	0.034	0.018
V_mic-DR_, cm^3^g^−1^	-	0.181	0.067	0.236	0.229
V_mic-HK_, cm^3^g^−1^	-	0.176	0.062	0.239	0.229
D_max-HK_, nm	-	0.53	1.09	0.46	0.49

**Table 5:** Results of I_a_, I_c_ and R_ct_ values.

Electrode	I_a_ (µA)	I_c_ (µA)	I_a_/I_c_	∆E_p_ (mV)	R_ct_ (kΩ)
CPE1	8.78 ± 0.31	−8.18 ± 0.29	1.07	287	/
CPE2	18.60 ± 0.65	−17.60 ± 0.62	1.05	217	25.61 ± 0.90
CPE3	30.00 ± 1.05	−26.00 ± 0.91	1.15	202	28.67 ± 1.00
CPE4	48.00 ± 1.68	−47.00 ± 1.64	1.02	202	11.18 ± 0.39
CPE5	25.70 ± 0.90	−24.45 ± 0.85	1.05	312	25.31 ± 0.88
CPE6	34.00 ± 1.19	−33.00 ± 1.15	1.03	287	26.30 ± 0.92
CPE7	31.00 ± 1.08	−30.00 ± 1.05	1.03	277	21.09 ± 0.74
CPE8	15.62 ± 0.55	−14.55 ± 0.51	1.07	121	25.02 ± 0.88

**Table 6:** Comparison of electrochemical parameters of various CPEs modified with Bi_2_O_3_.

Electrode	I_a_ (µA)	I_c_ (µA)	R_ct_ (kΩ)	Literature
GCP@Bi_2_O_3_	29.50	−22.4	37.40	[81]
Biochar-CB/PLA	13.20	−12.80	1.01	[82]
Bi_2_O_3_(n)@SWCNT/CP	36.60	−36.00	29.00	[83]
CAR_ALD/Bi_2_O_3_-Sm	48.00	−47.00	11.18	This manuscript

## Data Availability

The original contributions presented in this study are included in the article. Further inquiries can be directed to the corresponding author.
